# Forward Head Posture and Trunk Muscle Activation Patterns During a Rapid Leg-Raise Task in Asymptomatic Young Adults: A Cross-Sectional Study

**DOI:** 10.3390/jcm15103657

**Published:** 2026-05-09

**Authors:** Ibrahim M. Moustafa, Maryam M. Abdellatif, Monica Raja Kumari Raghunathan, Khadija Darwish, Zahra I. Almohsen, Hessa K. Alketbi, Noura I. Alfaraji, Iman Khowailed, Aliaa A. Diab, Deed E. Harrison

**Affiliations:** 1 Department of Physiotherapy, College of Health Sciences, University of Sharjah, Sharjah 27272, United Arab Emirates; iabuamr@sharjah.ac.ae (I.M.M.); u21107334@sharjah.ac.ae (Z.I.A.);; 2 Neuromusculoskeletal Rehabilitation Research Group, RIMHS–Research Institute of Medical and Health Sciences, University of Sharjah, Sharjah 27272, United Arab Emirates; 3 Faculty of Physical Therapy, Cairo University, Giza 12613, Egypt; 4 CBP Nonprofit (A Spine Research Foundation), Eagle, ID 83616, USA

**Keywords:** forward head posture, anticipatory postural adjustments, feedforward activation, muscle co-contraction

## Abstract

**Background/Objectives**: Forward head posture (FHP) has been associated with alterations in cervical sensorimotor function; however, its relationship with trunk muscle activation during dynamic movement tasks remains incompletely understood. This study examined whether individuals with FHP demonstrate differences in the timing and magnitude of trunk muscle activation during a rapid lower-limb movement task. **Methods**: One hundred asymptomatic young adults (18–25 years) were classified as having normal head posture (NHP; craniovertebral angle (CVA) > 55°) or forward head posture (FHP; CVA < 50°) using PostureScreen^®^ Mobile. Surface electromyography (EMG) was recorded bilaterally from the external oblique (EO), lumbar multifidus (MF), and the transversus abdominis/internal oblique region (TrA/IO) during ten externally cued right-leg raises. EMG amplitude and onset latency relative to rectus femoris activation were extracted for each muscle. Two multivariate analyses of variance (MANOVA) assessed overall group differences in EMG amplitudes and onset latencies. Significant multivariate effects were followed by univariate ANOVAs. Group × Side mixed-model ANOVAs evaluated side-to-side activation patterns. Pearson correlation and regression analyses examined correlations between craniovertebral angle (CVA) and EMG variables. **Results**: MANOVA revealed significant overall differences between the FHP and NHP groups for EMG amplitude (Wilks’ λ = 0.20, F(6, 93) = 62.14, *p* < 0.001, η^2^p = 0.80) and onset latency (Wilks’ λ = 0.10, F(6, 93) = 133.73, *p* < 0.001, η^2^p = 0.90). Follow-up ANOVAs showed significant differences for all EMG variables (all *p* < 0.001), with large effect sizes (Cohen’s d = 1.0–3.2). Mixed-model ANOVAs demonstrated significant Group × Side interactions (all *p* < 0.05). CVA showed significant moderate to strong correlations with EMG amplitude and onset latency measures (r = 0.43–0.79). **Conclusions**: FHP was associated with later trunk muscle activation and altered EMG activation patterns during the leg-raise task, including reduced activity recorded from the TrA/IO region and increased EO activation. These findings suggest FHP is associated with different trunk neuromuscular activation strategies during dynamic tasks. Despite consistent CVA–EMG associations, extreme multicollinearity limits the interpretation of CVA as an independent predictor.

## 1. Introduction

Feedforward activation is a critical anticipatory mechanism in human motor control. It serves as the cornerstone of postural stability, enabling precise coordination between proximal stabilization and distal movement execution. This pre-programmed neuromuscular strategy, orchestrated by the central nervous system (CNS), ensures early activation of core stabilizers (e.g., transversus abdominis (TrA), multifidus, and quadratus lumborum) prior to limb motion, counteracting destabilizing forces and safeguarding spinal integrity during dynamic tasks [1,2,3]. Pioneering electromyographic studies reveal that trunk musculature activates within 50 ms preceding deltoid engagement, underscoring the CNS’s predictive capacity to optimize movement efficiency and mitigate injury risk [1,2,4]. Yet, emerging evidence suggests this finely tuned system hinges on uninterrupted proprioceptive input, a vulnerability exposed in populations with sensorimotor deficits [5,6,7].

Proprioceptive input plays an essential role in regulating the timing and magnitude of muscle activation during postural and movement tasks by contributing to the continuous updating of internal motor control models [6,8]. Alterations in afferent signaling—such as those associated with chronic pain, deafferentation, or sustained postural deviations—have been reported to influence anticipatory postural adjustments (APAs) and are associated with delayed activation of stabilizing muscles and increased mechanical loading on the spine [5,7,9,10]. For example, sustained neck flexion has been shown to modify cervical erector spinae activation timing during rapid arm movements, with delays of approximately 20–40 ms observed in both experimental settings and individuals with chronic neck pain [6,11].

Collectively, these findings suggest that cervical posture may be associated with changes in sensorimotor control processes involved in both reactive and anticipatory motor strategies. However, the extent to which these associations influence trunk muscle activation during dynamic tasks remains unclear [12,13].

Forward head posture (FHP) is a common postural deviation observed in both asymptomatic individuals and those with neck disorders. Previous studies have reported associations between FHP and alterations in sensorimotor function, including changes in cervical mechanoreceptor input, reduced joint position sense, and modifications in corticomotor excitability [11,14,15,16]. These findings suggest that sustained cervical postural deviations may be associated with altered sensorimotor integration processes involved in maintaining postural stability and coordinating movement responses to perturbations.

Although corrective interventions have been shown to improve proprioceptive accuracy and aspects of postural control in individuals with FHP [14,15], the extent to which FHP is associated with changes in the timing and magnitude of trunk muscle activation during rapid limb movements remains unclear. Addressing this question may provide further insight into whether postural alignment is associated with differences in neuromuscular activation strategies contributing to trunk stabilization during dynamic tasks.

Importantly, prior studies examining forward head posture have primarily focused on cervical sensorimotor control, postural stability, and regional muscle activation [11,14,15], whereas direct investigation of anticipatory trunk muscle activation during perturbation tasks in individuals with FHP remains limited. In parallel, the broader literature on anticipatory postural adjustments has commonly utilized upper-limb movement paradigms to assess feedforward trunk activation [1,4]. Less is known about how these mechanisms operate during lower-limb perturbations, which impose greater demands on lumbopelvic stability and intersegmental coordination [2]. Therefore, examining trunk muscle activation during a rapid single-leg raise task may provide additional insight into neuromuscular coordination strategies associated with FHP under functionally relevant conditions. Furthermore, investigating these relationships in asymptomatic individuals allows identification of posture-related neuromuscular differences independent of pain-related adaptations [12].

This gap is clinically significant, as delayed or reduced recruitment of the TrA and lumbar multifidus (LM) may lead to suboptimal spinal stability, increased risk of chronic pain, and impaired functional performance. By investigating how FHP affects anticipatory activation of core stabilizers, this study aims to advance understanding of the neurophysiological mechanisms underlying postural dysfunction and to inform targeted rehabilitation strategies focusing on both cervical alignment and deep trunk muscle control. This study tests the hypothesis that compared to normal posture, participants with FHP will have a difference in anticipatory activation of core trunk muscle stabilizers, reflected by delayed latency and increased EMG amplitude.

## 2. Materials and Methods

### 2.1. Study Design and Setting

A cross-sectional, case–control investigation was conducted in a controlled lab setting at the University of Sharjah, United Arab Emirates, after obtaining ethical approval from the Research Ethics Committee of the University of Sharjah (Ethical approval number: REC-25-01-23-01-PG, 15 May 2025). Participants were purposively sampled from University of Sharjah students and screened for eligibility via an online questionnaire and subsequent physical assessment. Informed consent was obtained from all participants. Data was collected from 19 May to 25 November 2025.

### 2.2. Participants

One hundred healthy young adults, aged between 18 and 25 years, were recruited for this study. Participants were stratified into two groups based on their craniovertebral angle (CVA), as measured by the PostureScreen^®^ Mobile application (PSM) (Posture Co, Inc.; Trinity, FL, USA). To ensure clear classification and reduce misclassification near the threshold, a 5° buffer zone between cases with FHP and controls without FHP was introduced. Accordingly:

- FHP: CVA < 50°
- Normal head posture (NHP): CVA > 55°

Participants with intermediate CVA values (50–55°) were excluded from group comparisons to enhance between-group discrimination and minimize overlap. This stratification approach is consistent with the prior literature recognizing postural transition zones [17]. The PSM is a reliable and validated tool for static seated and standing postural assessment [18,19].

Purposive sampling was used to recruit participants who met predefined postural criteria, allowing clear classification into forward head posture and normal posture groups. This approach improved group separation and reduced misclassification, but it may introduce selection bias and limit generalizability.

### 2.3. Eligibility Criteria

- **Inclusion Criteria:**
  ○ Age between 18 and 25 years.
  ○ No current or prior history of spine, neurological, or orthopedic disorders.
  ○ No history of spine or lower-limb surgery.
  ○ Eligibility required right-leg dominance, determined by the leg the participant would use to kick a ball. This standardized limb dominance to reduce variability and avoid confounding in EMG onset and amplitude measures.
- **Exclusion Criteria:**
  ○ Neurological conditions.
  ○ Musculoskeletal disorders.
  ○ Systemic conditions that affect movement.
  ○ Presence of pain or injury.
  ○ Pregnancy.

### 2.4. Instrumentation

**Figure 1:**
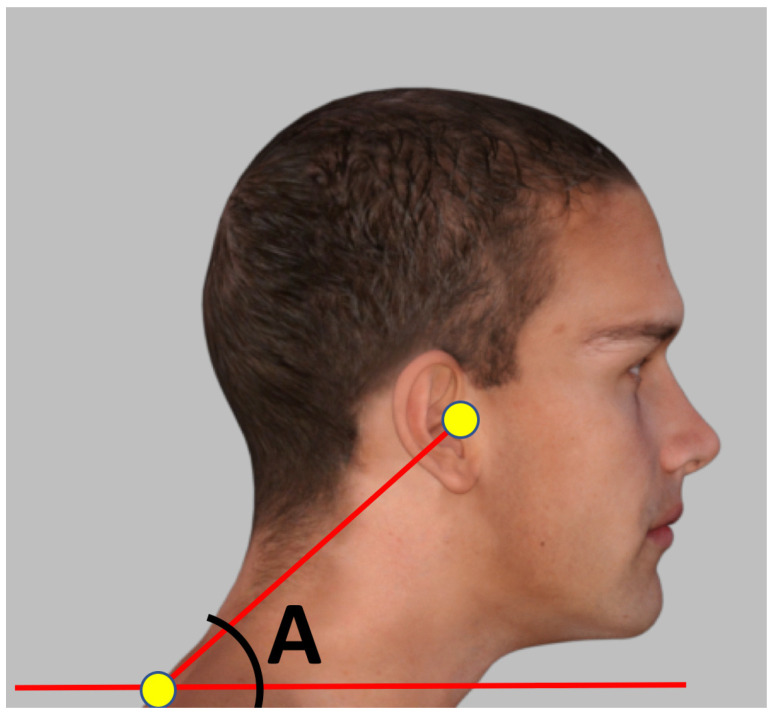
The CVA measurement with the PostureScreen Mobile App Posture Co, Inc.; Trinity, FL, USA). The CVA (angle A) is measured by constructing a line from the C7 spinous process to the tragus and quantifying the angle between this line and the true horizontal. Shown is an animated image of a human to represent the CVA as used in this investigation.

- **Posture Assessment:** PSM captured four standard photographic views: anterior, posterior, right lateral, and left lateral. Use of both the right and left lateral views in PSM averages out any possible left-to-right differences for the CVA, accounting for possible left/right head rotation projection errors. Anatomical landmarks (tragus of the ear, acromion process, and C7 spinous process) were digitally marked for CVA calculation (Figure 1).
- **EMG:** NeXus Q32 system (MindMedia NeuroLOGX B, Roermond, The Netherlands) was used to capture surface EMG signals to quantify the feedforward onset latency and amplitude prior to the externally cued, self-executed perturbation task. The system provided high-fidelity signal acquisition with a sampling rate of 2048 Hz and real-time monitoring capabilities.

#### 2.4.1. EMG Muscle Selection and Placement

Surface electromyography (sEMG) electrodes were placed according to SENIAM guidelines and established anatomical references [20]. Transversus abdominis/internal oblique (TrA/IO): the placement used was 2 cm medially relative to the anterior superior iliac spine (ASIS). Surface EMG recordings from this site reflect the combined activity of the TrA/IO region and do not permit the selective isolation of the transversus abdominis.

1. LM: 2 cm lateral to the midline at the L4 spinous process level.
2. External oblique (EO): midway between the iliac crest and the costal margin along the mid-axillary line, angled slightly oblique.

LM electrodes were placed according to SENIAM guidelines, and TrA/IO and EO electrodes followed the protocol described by Lin et al. [21]. Electrodes were aligned with muscle fiber direction to optimize signal quality and reduce noise. The ground electrode was placed on the right iliac crest. Before application, the skin was cleaned with alcohol and allowed to dry to improve adhesion and reduce impedance.

#### 2.4.2. Definition of Movement Onset

Leg-raise onset (T_0_) was defined a priori as the onset of rectus femoris (RF) EMG activity in the raised (right) limb. RF onset was identified from the full-wave-rectified, 50 ms RMS-smoothed RF signal as the first sample exceeding the mean + 3 SD of a −400 to −200 ms pre-cue baseline and remaining above the threshold for ≥25 ms. A 150 ms refractory period was applied to prevent multiple detections within a trial. Two raters blind to group placement verified all detections and disagreement was solved by agreement. Trunk-muscle latencies are reported relative to RF onset (negative values indicate anticipatory activation).

#### 2.4.3. Electrode Placement and Crosstalk Mitigation

To reduce crosstalk between the hip flexors and abdominal wall, TrA/IO electrodes were placed 2 cm medial to the ASIS and verified by palpation during gentle abdominal hollowing to confirm local activation without hip-flexor spillover. Inter-electrode spacing was standardized at 20 mm, and leads were secured and routed caudally to minimize cable movement. A ground electrode was placed on the right iliac crest to improve common-mode rejection. Participants practiced isolated TrA/IO hollowing and brief resisted hip flexion, while channels were monitored in real time to ensure RF activity did not produce synchronous TrA/IO bursts beyond baseline noise.

### 2.5. Experimental Procedure

Participants stood barefoot on a level surface with feet shoulder-width apart, arms relaxed at their sides, and their gaze fixed on an eye-level visual target. They completed 10 trials of an externally cued, self-initiated rapid right single-leg raise:

- At the first auditory cue, participants flexed the right hip to 90° as quickly as possible.
- At the second auditory cue, participants slowly returned to the starting position.

Participants were instructed to avoid compensatory trunk movements. Trials were separated by 30 s of rest to minimize fatigue, and the interval between auditory cues varied to reduce anticipation. Participants completed up to 10 practice trials before data collection to standardize performance and limit learning effects.

During the externally cued, self-executed perturbation task, sEMG recorded bilateral activity from TrA/IO, LM, and EO. These muscles were selected because they are key contributors to anticipatory postural adjustments (APAs) and help stabilize the spine via feedforward control during voluntary limb movements [22].

#### sEMG Amplitude Normalization

Surface electromyography (sEMG) signals were normalized to facilitate comparisons of muscle activation amplitudes within and across participants during the task. Normalization was performed using maximal voluntary isometric contraction (MVIC) procedures for each muscle region, following commonly used electromyographic normalization practices and adapted to the muscles examined (external oblique [EO], lumbar multifidus [LM], and the TrA/IO).

MVIC reference contractions were obtained using standardized manual muscle testing (MMT) positions designed to preferentially activate each muscle group while maintaining consistent joint positions and resistance application.

TrA/IO region: Participants performed abdominal hollowing in crook lying, gently drawing in the abdominal wall while minimizing pelvic and rib-cage movement. This maneuver was used to preferentially activate the deep abdominal region recorded by the TrA/IO region electrode placement.
LM: Participants lay prone and performed a contralateral arm-and-leg lift while keeping the lumbar spine neutral.
EO: Participants performed an isometric side-lying trunk flexion against manual resistance applied just above the iliac crest.

Each contraction was sustained for 5 s and was repeated 3 times, using 30–60 s of rest between trials to minimize fatigue. For each muscle and side, the highest root mean square (RMS) achieved in the MVIC trials was used as the reference value representing 100% MVIC. EMG amplitude recorded during the experimental trials was subsequently expressed as a percentage of this MVIC reference (%MVIC). Recognizing the known challenges associated with achieving maximal activation of deep trunk muscles during isolated MVIC tasks, several procedures were implemented to enhance consistency and reliability:

- Consistent verbal encouragement was provided during all MVIC trials to promote maximal effort.
- For the TrA/IO region, abdominal hollowing in crook lying was used as a standardized reference maneuver; however, this task does not represent a true maximal voluntary contraction. Therefore, normalized amplitude values (%MVIC) for this region should be interpreted as relative measures referenced to a consistent submaximal task rather than absolute indicators of maximal muscle activation.
- Pilot testing was conducted on a subset of participants prior to the main data collection to verify the feasibility and consistency of the MVIC procedures.

Previous studies have reported that MVIC-based normalization of trunk muscle sEMG signals demonstrates acceptable intra-person reliability and reduces inter-person variability when standardized procedures are applied [23]. Accordingly, electrode placement, instructions, and rest intervals were standardized to improve reproducibility and reduce measurement variability.

### 2.6. EMG Signal Acquisition and Processing

Surface electromyography (sEMG) was recorded at 2048 Hz using a NeXus Q32 system (MindMedia NeuroLOGX B, Netherlands). During acquisition, a 20–450 Hz hardware band-pass filter was applied to reduce movement artifacts and electrical noise while retaining physiologically relevant EMG frequencies.

Skin was shaved as needed, lightly abraded, and cleaned with alcohol to reduce electrode–skin impedance to <10 kΩ. Bipolar Ag/AgCl surface electrodes were placed according to SENIAM recommendations [20] (when applicable) and established anatomical guidelines for trunk muscles, aligned with the muscle fiber direction, with a 20 mm center-to-center inter-electrode distance to minimize crosstalk.

#### 2.6.1. Signal Preprocessing

Raw EMG signals were initially smoothed using a Savitzky–Golay filter (polynomial order = 4, frame length = 21 samples) to reduce high-frequency noise while preserving the temporal characteristics of the signal, particularly around activation onset. The signals were then full-wave rectified and RMS-smoothed with a 50 ms moving window to generate the amplitude envelope for subsequent analyses.

The window of 50 ms RMS was selected to stabilize baseline fluctuations and improve the reliability of onset detection. Pilot testing indicated that this window produced onset estimates comparable to those obtained using shorter smoothing windows, with differences of less than 5 ms.

#### 2.6.2. Onset Detection

Trunk muscle onset timing (EO, LM, and TrA/IO) was determined with an integrated profile (IP) methodology, which has been previously applied in trunk muscle EMG analysis [24]. For each muscle, the processed EMG signal (rectified and RMS-smoothed) was cumulatively integrated within a detection window extending from −150 ms to +200 ms relative to rectus femoris (RF) onset. Muscle onset was defined as the time point corresponding to the greatest vertical deviation between the integrated EMG curve and a linear line connecting the start and end of the integration window, representing the point of greatest change in signal slope.

Onset latency was calculated as:

$$Latency\;\left(ms\right)=Trunk\;Muscle\;Onset\;Time-RF\;Onset\;Time$$

Negative values indicate anticipatory (feedforward) activation before RF onset. Latencies were calculated for each trial and averaged across the 10 perturbation trials for each muscle and participant.

#### 2.6.3. Amplitude Analysis

The amplitude of muscle activation was calculated from the RMS-smoothed EMG envelope within a peri-movement window extending from −50 ms to +100 ms relative to rectus femoris (RF) onset. This time window was selected to capture trunk muscle activity occurring immediately before and shortly after the initiation of the prime mover, reflecting early neuromuscular responses associated with the movement task.

Normalization was performed separately for each muscle and side. The mean %MVIC from ten trials per participant was used for statistical analysis to enhance measurement stability and minimize within-subject variability.

$$Normalized\;EMG\;\left(\%MVIC\right)=\frac{Mean\;RMS\;during\;task\;window}{Peak\;RMS\;during\;MVIC}\times 100$$

Mean %MVIC for each muscle was calculated across the 10 trials to improve reliability and reduce within-subject variability.

### 2.7. Outcome Measures

The primary outcome variables were the onset latencies of trunk muscle activity relative to the onset of RF (ms) for EO, LM, and TrA/IO recorded on each side. Latency values that were negative indicated muscle activation occurring before RF onset (anticipatory activation). In contrast, positive latency values indicate activation following RF onset.

EMG amplitude was quantified as the mean RMS amplitude within the predefined peri-movement time window relative to RF onset and expressed as %MVIC. This measure reflects the magnitude of muscle activation during the movement task and provides information about the relative level of muscle recruitment during the analyzed period.

### 2.8. Sample Size Determination

Prior to study initiation, a power analysis was conducted using G*Power 3.1 for a mixed-design ANOVA (between–within interaction). Assuming α = 0.05, 80% power, and a Group × Side interaction effect size of Cohen’s f = 0.20 (from pilot data), with a repeated-measures correlation of 0.50 and a non-sphericity correction of 1.0, the required sample size was n = 100 (50 per group).

### 2.9. Statistical Analysis

All analyses were conducted using standard statistical software, with significance set at *p* < 0.05. Normality was assessed using the Shapiro–Wilk test and Q–Q plots. Homogeneity of variances between groups was evaluated using Levene’s test. Since the assumptions were satisfied, parametric statistical analyses were performed.

#### 2.9.1. Descriptive Statistics and Baseline Comparisons

Mean and standard deviation were calculated for all continuous variables. Baseline demographic and clinical characteristics (age, BMI, and CVA) were analyzed between the two groups using independent samples *t*-tests. A Chi-square (χ^2^) test was used to confirm the balanced distribution of gender between groups.

#### 2.9.2. Primary Between-Group Comparisons

Two separate one-way multivariate analyses of variance (MANOVA) were performed to assess overall group differences. The first MANOVA used the six EMG amplitude variables as dependent variables, and the second used the six EMG onset latency variables. Group (FHP vs. NHP) served as the independent variable. Wilks’ Lambda (λ) was used as the test statistic, and partial eta-squared (η^2^p) was calculated to determine the effect size. Significant MANOVA results were followed by univariate analyses of variance (ANOVA) for each dependent variable to identify specific group differences. Cohen’s d was calculated for each univariate comparison to quantify the magnitude of the difference.

#### 2.9.3. Side-to-Side Asymmetry Analysis

To investigate potential asymmetries, a series of 2 (Group: FHP, NHP) × 2 (Side: Left, Right) mixed-model ANOVAs was conducted for each muscle’s amplitude and onset latency data. The primary focus was on the Group × Side interaction effect, which would indicate that the pattern of side-to-side asymmetry differs between the FHP and NHP groups. To control the familywise Type I error rate, Holm–Bonferroni correction was applied to univariate tests after significant multivariate results.

#### 2.9.4. Correlation and Regression Analysis

Pearson’s correlation coefficients (r) were calculated to identify the direction and strength of a possible linear relationship between CVA and each of the 12 EMG variables across the entire sample. Subsequently, a series of hierarchical multiple linear regression analyses was performed to determine if CVA could independently predict EMG outcomes after controlling for potential confounders. In each model, age, BMI, and gender were entered in the first block, followed by CVA in the second block. The change in R^2^, total R^2^, and the standardized beta coefficient (β) for CVA were reported. Statistical analyses were conducted using IBM SPSS Statistics for Windows, Version 29.0 (IBM Corp., Armonk, NY, USA).

## 3. Results

**Figure 2:**
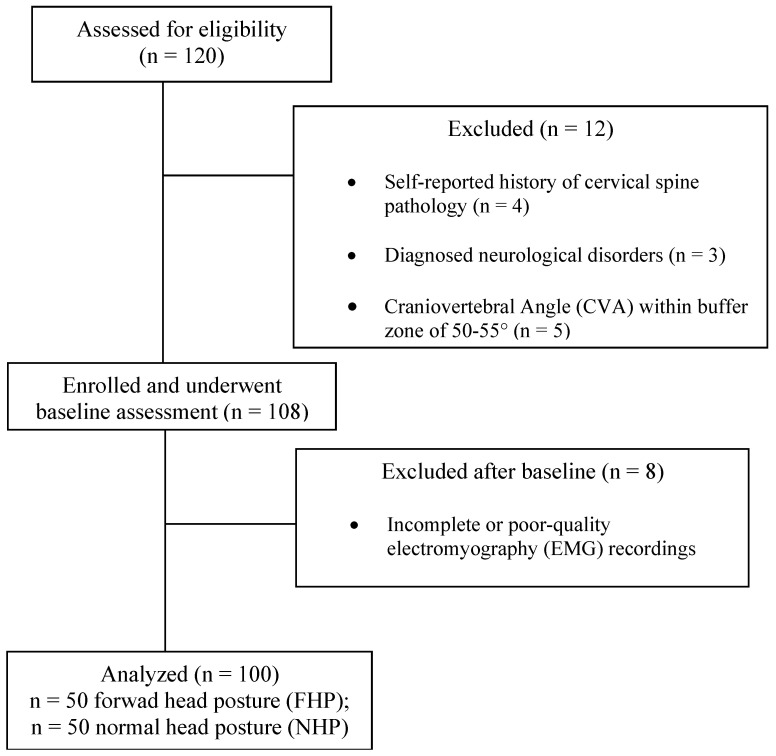
Participant flow chart showing recruitment, exclusions, and analysis.

A total of 120 volunteers were screened for eligibility. Twelve individuals were excluded prior to group allocation: four due to self-reported history of cervical spine pathology, three with diagnosed neurological disorders, and five whose CVA fell within the predefined buffer zone of 50–55°, established to avoid borderline classification between FHP and NHP. The remaining 108 eligible participants were enrolled and underwent baseline assessment. Eight of these were subsequently excluded due to incomplete or poor-quality EMG recordings, leaving a final analyzed sample of 100 participants (50 FHP, 50 NHP) with complete datasets and no missing values for any outcome measure (Figure 2). This process ensured that buffer zone exclusions were applied prospectively and that final group sizes were achieved without selective data removal that could bias comparisons.

### 3.1. Descriptive Statistics and Baseline Comparisons

The demographic and clinical characteristics of the two groups are presented in Table 1. There were no significant differences between the FHP and NHP groups in terms of age (*p* > 0.05) or BMI (*p* > 0.05), as visualized in Table 1. The gender distribution was perfectly matched by design (χ^2^(1) = 0, *p* = 1.0). As expected, there was a highly significant difference in CVA, with the FHP group demonstrating a much smaller angle than the NHP group (t(98) = −23.15, *p* < 0.001, Cohen’s d = −4.63).

### 3.2. Primary Between-Group Comparisons

The MANOVA for EMG amplitude revealed a significant overall difference between the FHP and NHP groups (Wilks’ λ = 0.20, F(6, 93) = 62.14, *p* < 0.001), with a large effect size (η^2^p = 0.80). Similarly, the MANOVA for EMG onset latency showed a highly significant overall group difference (Wilks’ λ = 0.10, F(6, 93) = 133.73, *p* < 0.001), with an extremely large effect size (η^2^p = 0.90).

The large effect sizes observed (η^2^p = 0.80–0.90) likely reflect the combination of a purposive-groups design, low within-group variability, and the use of multivariate analysis capturing shared variance across correlated EMG variables. These values should therefore be interpreted as reflecting strong group separation within the study sample rather than population-level effect estimates.

**Figure 3:**
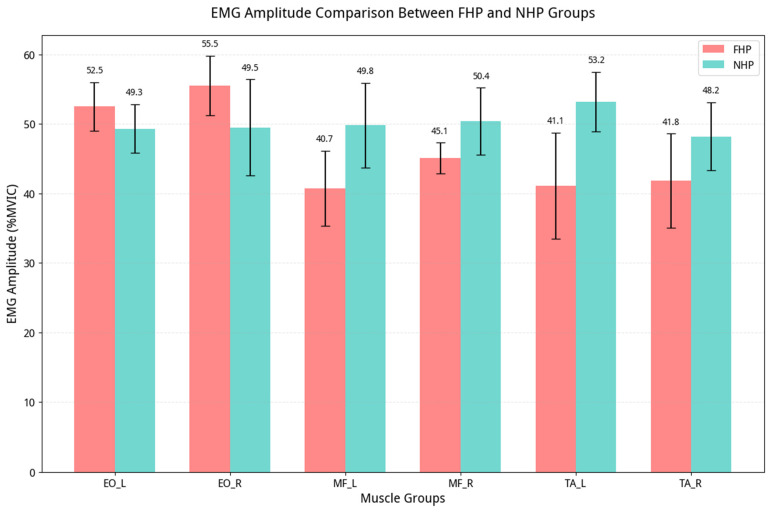
Combined grouped bar chart comparison of EMG amplitude (%MVIC) for all muscle pairs (EO, MF, and TA) between the FHP and NHP groups (Mean ± SD).

**Figure 4:**
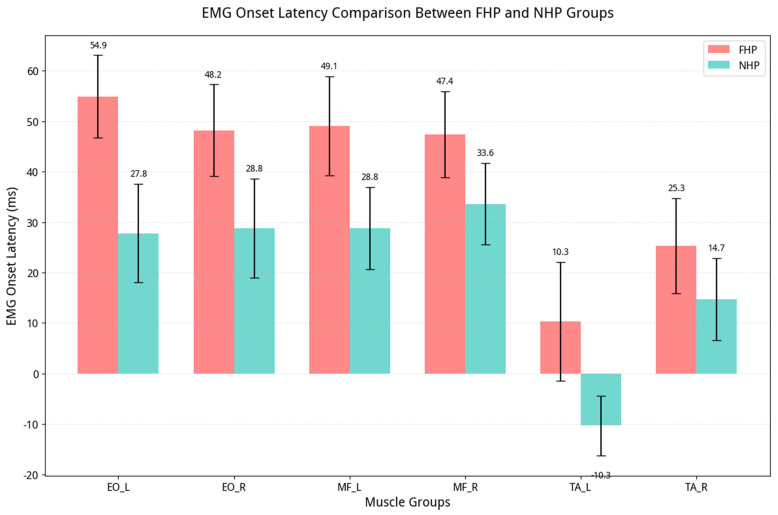
Combined grouped bar chart comparison of EMG onset latency (ms) for all muscle pairs (EO, MF, and TrA/IO region) between the FHP and NHP groups (Mean ± SD).

Follow-up univariate ANOVAs confirmed that all 12 individual EMG variables were significantly different between the groups (all *p* < 0.001), with all effect sizes being large (Cohen’s d ranging from 1.0 to 3.2). A summary of these univariate results is presented in Table 2. The group differences are visualized in the following plots, which are representative examples for all 12 EMG variables (Figure 3 and Figure 4).

### 3.3. Side-Dependent Activation Patterns

**Figure 5:**
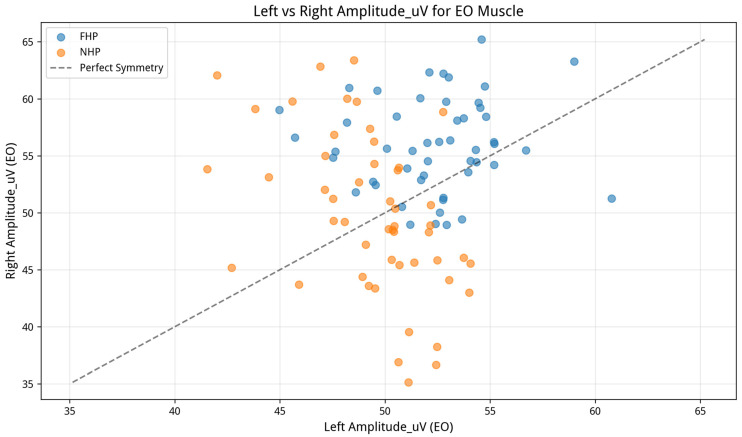
Representative interaction plot comparing Left vs. Right EO amplitude, showing different muscle symmetry patterns between the FHP and NHP groups.

To investigate differential asymmetry patterns, a series of 2 (Group: FHP, NHP) × 2 (Side: Left, Right) mixed-model ANOVAs was conducted for each muscle’s amplitude and onset latency data. The analyses revealed significant Group × Side interaction effects for all six EMG variable pairs: external oblique (EO) amplitude (F(1, 195) = 5.60, *p* = 0.018, η^2^p = 0.028), EO onset (F(1, 195) = 10.88, *p* = 0.001, η^2^p = 0.053), lumbar multifidus (MF) amplitude (F(1, 195) = 12.32, *p* < 0.001, η^2^p = 0.059), MF onset (F(1, 195) = 10.90, *p* < 0.001, η^2^p = 0.053), transversus abdominis/internal oblique (TrA/IO region) amplitude (F(1, 195) = 12.17, *p* < 0.001, η^2^p = 0.059), and TrA/IO region onset (F(1, 195) = 19.00, *p* < 0.001, η^2^p = 0.089). These results indicate that the pattern of left–right trunk muscle activation during the standardized right-leg perturbation task differed significantly between the FHP and NHP groups. As visualized for EO amplitude in Figure 5, this suggests that FHP is associated with altered bilateral coordination during the dynamic task.

### 3.4. Correlation and Regression Analysis

**Figure 6:**
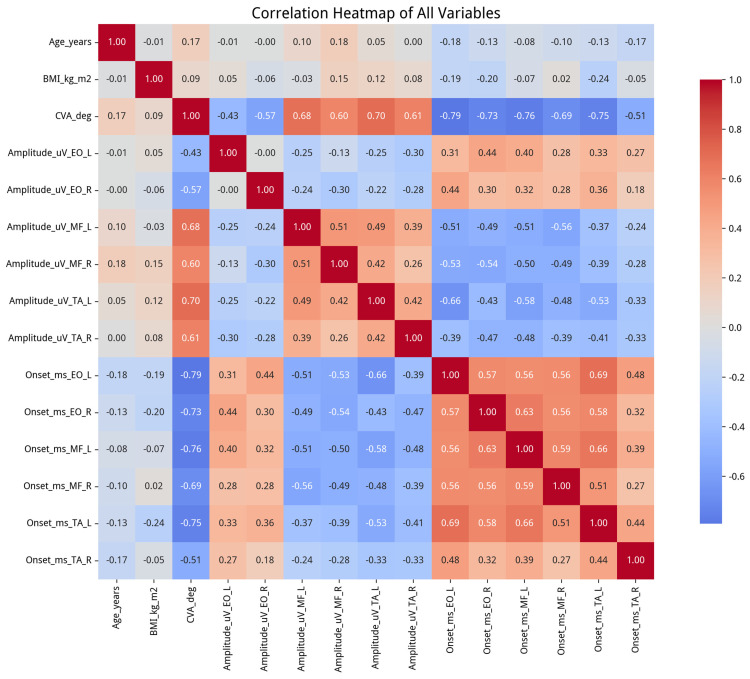
Comprehensive correlation heatmap of all numeric variables, highlighting strong correlations between CVA and EMG measurements, especially onset times.

To evaluate the relationship between head posture and neuromuscular activation patterns, Pearson’s correlation coefficients (r) were calculated to assess the strength and direction of the linear association between the craniovertebral angle (CVA) and each of the 12 EMG variables across the entire sample. As shown in the comprehensive heatmap (Figure 6), CVA demonstrated statistically significant correlations with all EMG variables. The strongest relationships were observed with onset latency variables, particularly on the left side, with correlation coefficients ranging from r = −0.73 to −0.79 (all *p* < 0.001). These findings suggest that smaller CVA values (i.e., more pronounced FHP) are associated with longer muscle activation onset latencies.

**Figure 7:**
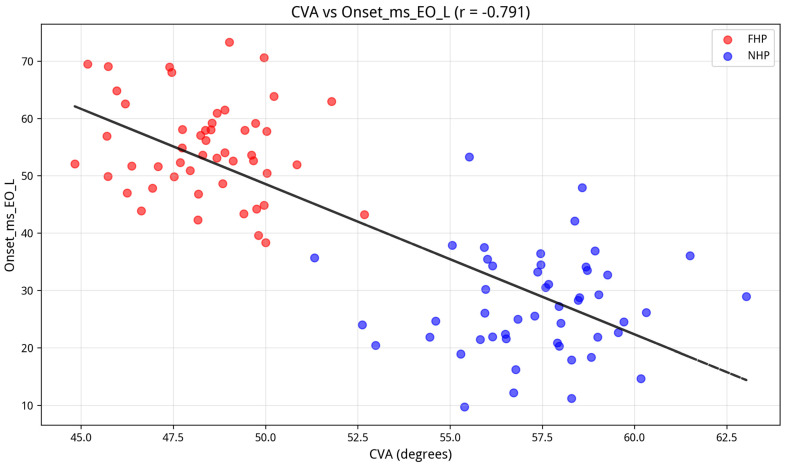
Representative scatter plot with regression line, showing the strong negative correlation between CVA and Left EO onset timing.

Furthermore, CVA showed moderate to strong positive correlations with amplitude variables for the lumbar multifidus (MF) and the transversus abdominis/internal oblique (TrA/IO) region (r = 0.60 to 0.70), and moderate negative correlations with external oblique (EO) amplitude (r = −0.43 to −0.57). The scatter plot in Figure 7 provides a visual representation of one of these relationships.

The regression analyses demonstrated that CVA was significantly associated with all EMG outcomes after adjustment for age, BMI, and gender. However, these findings should be interpreted with caution due to substantial multicollinearity among predictors (maximum VIF = 92.83), which may inflate standard errors, destabilize regression coefficients, and limit the ability to attribute independent effects to individual predictors. Therefore, the results are best interpreted as reflecting associative relationships within a set of correlated variables rather than independent predictive effects of CVA.

To determine if CVA was associated with EMG outcomes after controlling for potential confounders, a series of hierarchical multiple linear regression analyses was performed. In each of the 12 models, age, BMI, and gender were entered in the first block, followed by CVA in the second block. The results for the second block are detailed in Table 3. The analyses showed that CVA was significantly correlated with all 12 EMG outcomes after adjustment for age, BMI, and gender (all *p* < 0.001). The inclusion of CVA significantly increased the explained variance for all models, with a change in R-squared (ΔR^2^) values ranging from 0.198 to 0.574. However, these regression findings should be interpreted cautiously because of high multicollinearity between predictors (maximum VIF = 92.83), which can affect the stability of the regression coefficients. Despite this, the consistent, strong correlation of CVA across all models underscores its predictive relationship with neuromuscular function.

## 4. Discussion

This study investigated the association between forward head posture (FHP) and trunk muscle activation patterns during a dynamic lower-limb movement task. We hypothesized that participants with FHP would demonstrate differences in anticipatory activation of trunk stabilizing muscles, reflected by delayed onset latency and increased EMG amplitude. The findings partially support this hypothesis, as participants with FHP demonstrated significantly delayed onset timing of trunk muscles, including the lumbar multifidus (LM) and the transversus abdominis/internal oblique (TrA/IO) region, compared to participants with normal head posture (NHP). However, contrary to our expectations, reduced normalized activation amplitudes were observed in the TrA/IO region and LM, whereas the external oblique (EO) exhibited higher activation levels during the analyzed time window in the FHP group.

These findings suggest differences in trunk neuromuscular activation patterns between posture groups during the movement task. In general, our results are consistent with previous studies reporting associations between cervical postural deviations and alterations in sensorimotor control and trunk muscle activation during functional movements [1,2,5,9,14]. While strong associations were observed between CVA and EMG variables, these findings should be interpreted in light of the study design, which intentionally maximized group separation and may have contributed to the magnitude of these relationships. Although CVA showed consistent associations with EMG variables, the presence of extreme multicollinearity limits the ability to interpret CVA as an independent predictor. These findings should therefore be understood as reflecting shared variance within a correlated set of variables rather than distinct predictive contributions.

### 4.1. Trunk Muscle Activation Timing

Anticipatory postural adjustments (APAs) represent preparatory neuromuscular responses that occur before or near the onset of voluntary limb movement and contribute to maintaining trunk and pelvic stability during dynamic tasks [25,26]. In healthy individuals, trunk muscles such as the lumbar multifidus (LM) and the deep abdominal muscle region recorded from the transversus abdominis/internal oblique (TrA/IO region) electrode site are typically activated shortly before or around the onset of prime mover activity during rapid limb movements [4,25]. It should be noted that surface EMG recordings from this region reflect combined TrA/IO region activity and do not permit selective measurement of the transversus abdominis.

In the present study, participants with FHP demonstrated a later onset timing of trunk muscle activation compared with individuals with NHP. These differences were observed across both the deep trunk muscle regions (LM and TrA/IO region) and the more superficial EO, suggesting that cervical postural alignment may be associated with differences in trunk muscle activation timing during dynamic movement tasks. Similar alterations in trunk muscle activation timing have been reported in populations with chronic neck or low back pain, where delayed trunk muscle responses during limb movements have been interpreted as changes in neuromuscular control strategies [1,22,27,28].

An additional observation was that the magnitude of the onset delay in the FHP group was consistent across the examined trunk muscles, with differences ranging from approximately 15 to 23 ms for EO, LM, and the TrA/IO region. This relatively uniform delay pattern suggests that the observed differences were not limited to a single muscle group but involved multiple trunk muscles contributing to postural stabilization. Although the present study cannot determine the underlying mechanisms responsible for these timing differences, previous research has suggested that cervical postural deviations may be associated with changes in sensorimotor integration processes that contribute to the coordination of trunk muscle activity during movement [7,10,16].

Interestingly, increased activation amplitude of the EO muscle was observed alongside the delayed activation of the deeper trunk muscle regions. This pattern may reflect differences in trunk muscle recruitment during the movement task; however, the present study cannot determine whether this represents a compensatory response or simply an alternative activation strategy. Further studies incorporating additional biomechanical or neurophysiological measurements would be needed to clarify the mechanisms underlying these activation patterns.

### 4.2. Differences in Trunk Muscle Activation Amplitude

The amplitude results showed reduced normalized EMG activity recorded from the deep abdominal muscle region (TrA/IO region) and lumbar multifidus (LM) in participants with forward head posture (FHP), whereas the external oblique (EO) exhibited higher activation levels during the analyzed time window. These findings indicate differences in trunk muscle recruitment patterns between individuals with FHP and those with normal head posture (NHP) during the movement task. The observation of increased EO activation alongside reduced activity in the deeper trunk muscle regions is consistent with previous models of trunk muscle coordination suggesting that alterations in activation between deep and superficial trunk muscles may occur during tasks that challenge postural control or rapid movement responses [16,25]. However, the present study cannot determine whether the observed pattern represents a compensatory response, an alternative recruitment strategy, or task-specific motor behavior. Furthermore, our findings should be interpreted cautiously, as normalization of the TrA/IO region signal was based on a submaximal reference task, and surface EMG recordings reflect regional muscle activity rather than isolated deep muscle activation.

An additional observation was that EO activation amplitude increased in the FHP group despite a later activation onset relative to the NHP group. Because the EO is a superficial trunk muscle involved in generating trunk torque and contributing to global trunk movement, its increased activation during the analyzed period may reflect greater involvement of this muscle during the movement task. Nevertheless, the combination of increased amplitude and later onset timing should be interpreted cautiously, as the present study did not directly measure trunk kinematics, stability demands, or mechanical loading.

Similar trunk muscle activation patterns have been reported in studies examining balance tasks on unstable surfaces [26] and single-limb squats [2], where increased activation of superficial trunk muscles has been observed during tasks requiring greater postural control demands. These findings suggest that differences in postural alignment, such as forward head posture, may be associated with variations in trunk neuromuscular activation patterns during dynamic movements. However, further studies incorporating biomechanical and neurophysiological measurements are needed to clarify the mechanisms underlying these activation differences.

### 4.3. Potential Mechanisms

Several mechanisms may contribute to the differences in trunk muscle activation timing and amplitude observed in the present study.

#### 4.3.1. Cervical Sensorimotor Alterations

Previous research suggests that FHP may be associated with changes in cervical proprioceptive input and joint position sense [7,15,16]. Such alterations have been proposed to influence postural control and neuromuscular coordination during dynamic tasks. In the present study, reduced activation of the LM was observed bilaterally in participants with FHP, including on the stance side. During a unilateral leg-raise task, stance-side LM activity would typically contribute to resisting lumbar flexion moments and assisting in maintaining lumbopelvic stability. The FHP group’s bilateral LM activity reduction indicates altered trunk muscle recruitment rather than a solely task-specific response. Previous studies have also reported that cervical postural deviations can modify afferent feedback from cervical muscles and joints, potentially influencing postural control at other spinal segments, including the lumbar region [10,16,29,30].

These findings may reflect altered activation within the abdominal muscle region involved in trunk stabilization; however, interpretations regarding specific deep stabilizing muscles should be made cautiously due to the limited selectivity of surface EMG recordings.

#### 4.3.2. Central Sensorimotor Processing

Alterations in cervical alignment have been associated with changes in neurophysiological measures such as somatosensory evoked potentials and corticospinal conduction characteristics, with some studies reporting improvements following sagittal realignment interventions [13,31]. Although the present study did not directly assess corticospinal excitability, these findings suggest that cervical posture may be associated with broader sensorimotor processes involved in coordinating postural muscle activity.

#### 4.3.3. Interaction with Cognitive and Autonomic Factors

Forward head posture has also been linked to changes in autonomic regulation and increased cognitive–motor dual-task demands during postural control tasks [12,31,32]. Such factors may influence the timing and coordination of trunk muscle activation during dynamic movements, although these mechanisms were not directly examined in the current study. Taken together, these findings suggest that cervical postural alignment may be associated with differences in trunk neuromuscular activation patterns during movement tasks. However, the present study cannot determine the specific mechanisms underlying these differences, and further investigations incorporating biomechanical, neurophysiological, and sensorimotor assessments are needed to clarify these relationships.

### 4.4. Consistency and Contrasts with Previous Findings

The present findings are generally consistent with previous research reporting delayed activation of trunk muscles, including the LM and deep abdominal muscle regions, in individuals with spinal disorders or altered postural control [1,4,22]. Although the participants in the current study were asymptomatic, the observed differences in trunk muscle activation timing suggest that variations in postural alignment may also be associated with neuromuscular activation patterns during movement tasks.

Randomized controlled trials examining postural correction interventions have reported improvements in pain, function, and certain sensorimotor measures following restoration of cervical or thoracic alignment using extension traction or posture-corrective orthoses [11,15,31,33,34]. While these studies suggest that spinal alignment may influence sensorimotor function, the present study did not evaluate treatment effects and therefore cannot determine whether similar mechanisms explain the activation differences observed here.

Some previous studies have also reported inconsistent changes in trunk muscle activation timing following stabilization training alone, indicating that multiple factors—including posture, sensorimotor control, and task demands—may contribute to trunk muscle recruitment patterns [22,27]. These findings support the view that postural alignment and neuromuscular control may be interrelated, although the direction of this relationship remains unclear.

An additional observation in the present data was the difference in TrA/IO region activation symmetry patterns between the NHP and FHP groups during the right single-leg raise task. In the NHP group, a contralateral (left-dominant) activation pattern was observed in the TrA/IO region. Such contralateral recruitment has been described in studies examining trunk muscle coordination during rapid limb movements, where contralateral abdominal activation contributes to resisting pelvic rotation and assisting trunk stabilization in the transverse plane [25,35]. In contrast, the FHP group demonstrated a reduced contralateral bias and a tendency toward more symmetrical or ipsilateral activation patterns. This difference may indicate variations in trunk muscle recruitment strategies between posture groups during the task. However, the present study did not directly measure trunk kinematics, pelvic motion, or stability demands, and therefore, the functional implications of this activation pattern remain speculative.

### 4.5. Clinical Implications

From a clinical perspective, the present findings suggest that cervical postural alignment may be associated with differences in trunk muscle activation patterns during dynamic movement tasks. These observations highlight the potential relevance of considering postural alignment when evaluating neuromuscular control strategies in clinical and rehabilitation settings. Previous studies have reported improvements in pain, functional outcomes, and certain sensorimotor measures following interventions aimed at improving cervical and thoracic alignment [11,15,31,33,34]. However, the present study did not assess clinical outcomes or treatment effects; therefore, such findings should not be directly extrapolated to the current results.

Forward head posture is common among young adults, partly associated with prolonged device use and sedentary behavior [36]. In this context, identification of postural deviations may be relevant during clinical assessment, particularly when evaluating movement patterns and neuromuscular coordination. However, as the present study involved asymptomatic individuals, these findings should not be interpreted as evidence of clinical impairment or as directly related to pain. Overall, the findings may provide insight into posture-related variations in neuromuscular coordination that could be considered in assessment and preventive contexts. Longitudinal and interventional studies are required to determine whether modifying postural alignment influences trunk muscle activation patterns or clinical outcomes such as pain, balance, or physical performance. Accordingly, causal relationships cannot be inferred from the present findings.

### 4.6. Limitations and Future Directions

Several limitations should be considered when interpreting the findings of this study. The cross-sectional design precludes causal inference regarding the relationship between forward head posture (FHP) and trunk muscle activation. Longitudinal and interventional studies incorporating pre–post EMG assessments, proprioceptive measures, and dual-task paradigms are needed to clarify the directionality and mechanisms of these associations.

The use of purposive sampling may introduce selection bias and limit external validity. Accordingly, the findings may not be generalizable to populations with different age ranges, clinical conditions, or varying degrees of postural deviation. Craniovertebral angle (CVA) was used both to define postural groups and as a continuous variable in subsequent analyses, and participants with intermediate CVA values were excluded to enhance group separation. While this approach improves classification clarity and internal validity, it may amplify between-group differences and strengthen associations due to range restriction. Therefore, effect sizes and correlations should be interpreted with caution, and future studies including the full spectrum of CVA values are warranted.

The perturbation protocol was limited to a right-leg task. As such, the observed Group × Side effects cannot be interpreted as definitive evidence of bilateral asymmetry. Inclusion of matched left-leg conditions in future studies is necessary to distinguish generalized postural control differences from task-specific lateralized responses. The regression analyses were affected by substantial multicollinearity, as indicated by high variance inflation factors (VIFs). Under these conditions, regression coefficients may be unstable and should not be interpreted as independent effects. Future research should consider alternative approaches, such as dimension reduction or penalized regression techniques, to address collinearity.

Surface EMG has inherent limitations in assessing deep musculature, including limited spatial selectivity and potential crosstalk from adjacent muscles. In addition, recordings from the transversus abdominis/internal oblique (TrA/IO) region reflect combined regional activity rather than muscle-specific activation. Normalization of EMG amplitude for the TrA/IO region was based on abdominal hollowing in crook lying, which represents a standardized submaximal reference rather than a true maximal voluntary contraction. Consequently, absolute %MVIC values—particularly near movement onset—should be interpreted with caution. The amplitude findings are therefore more appropriately considered as relative between-group differences under consistent conditions rather than precise estimates of deep muscle function.

Future studies may expand on these findings by incorporating additional postural variables (e.g., thoracic kyphosis and three-dimensional head translation) and by applying advanced analytical approaches, including machine learning, to better characterize neuromuscular coordination patterns and their relationship to intervention outcomes.

## 5. Conclusions

Forward head posture (FHP) was associated with differences in trunk muscle activation timing and amplitude during a standardized rapid leg-raise task. Specifically, participants with FHP demonstrated a delayed onset of trunk muscles alongside a differential activation pattern characterized by reduced activity in the lumbar multifidus and TrA/IO region and increased reliance on the external oblique. Due to the high multicollinearity between predictors (maximum VIF = 92.83), our results are best interpreted as reflecting associative relationships within a set of correlated variables rather than independent predictive effects of cervical spine posture. However, given the cross-sectional design and methodological considerations, including the extreme-groups approach and EMG-related constraints, these results should be interpreted as associative rather than causal. Future longitudinal and interventional studies are required to determine whether modification of postural alignment influences trunk neuromuscular control and related functional outcomes.

## Figures and Tables

**Table 1:** Demographic data.

Variable	FHP Group (n = 50)	NHP Group (n = 50)	*p*-Value
Age (years)	21.26 ± 1.90	21.80 ± 1.60	>0.05
BMI (kg/m^2^)	22.16 ± 3.06	23.26 ± 3.08	>0.05
CVA (degrees)	48.36 ± 1.69	57.33 ± 2.16	<0.001
Gender (Female/Male)	25/25	25/25	1.000

**Table 2:** Comparison of EMG amplitude and onset latency between participants with forward head posture (FHP) and normal head posture (NHP).

EMG Variable	FHP (n = 50) Mean ± SD	NHP (n = 50) Mean ± SD	Mean Difference (95% CI)	Cohen’s d	*p*-Value *
**Amplitude (%MVIC)**					
EO—Left	52.5 ± 3.5	49.3 ± 3.5	3.2 [1.81, 4.59]	0.91	<0.001
EO—Right	55.5 ± 4.3	49.5 ± 6.9	6.0 [3.72, 8.28]	1.03	<0.001
LM—Left	40.7 ± 5.4	49.8 ± 6.1	−9.1 [−11.38, −6.82]	1.58	<0.001
LM—Right	45.1 ± 2.2	50.4 ± 4.8	−5.3 [−6.78, −3.82]	1.45	<0.001
TrA/IO region—Left	41.1 ± 7.6	53.2 ± 4.3	−12.1 [−14.55, −9.65]	1.96	<0.001
TrA/IO region—Right	41.8 ± 6.8	48.2 ± 4.9	−6.4 [−8.75, −4.05]	1.07	<0.001
**Onset Latency (ms)**					
EO—Left	54.9 ± 8.2	27.8 ± 9.8	27.1 [23.52, 30.68]	3.00	<0.001
EO—Right	48.2 ± 9.1	28.8 ± 9.8	19.4 [15.65, 23.15]	2.05	<0.001
LM—Left	49.1 ± 9.8	28.8 ± 8.1	20.3 [16.73, 23.87]	2.26	<0.001
LM—Right	47.4 ± 8.5	33.6 ± 8.1	13.8 [10.51, 17.09]	1.67	<0.001
TrA/IO region—Left	10.3 ± 11.8	−10.3 ± 5.9	20.6 [16.90, 24.30]	2.22	<0.001
TrA/IO region—Right	25.3 ± 9.4	14.7 ± 8.2	10.6 [7.10, 14.10]	1.20	<0.001

*p*-values remained significant after Holm–Bonferroni correction for multiple comparisons. EO: External Oblique; LM: Lumbar Multifidus; TrA/IO: Transversus Abdominis/Internal Oblique region. * indicates statistically significant differences.

**Table 3:** Hierarchical Regression Analysis Predicting EMG Variables from CVA.

EMG Variable	Δ*R*^2^	*F*-Change	β	95% CI for B	*p*-Value	Total *R*^2^
Amplitude_uV_EO_L	0.198	23.56	−0.434	[−0.432, −0.183]	<0.001	0.201
Amplitude_uV_EO_R	0.335	50.05	−0.571	[−0.994, −0.562]	<0.001	0.364
Amplitude_uV_MF_L	0.457	81.55	0.678	[0.810, 1.260]	<0.001	0.468
Amplitude_uV_MF_R	0.319	49.45	0.596	[0.359, 0.637]	<0.001	0.387
Amplitude_uV_TrA/IO region_L	0.479	90.36	0.699	[1.055, 1.602]	<0.001	0.497
Amplitude_uV_TrA/IO region_R	0.380	58.81	0.611	[0.647, 1.091]	<0.001	0.387
Onset_ms_EO_L	0.574	152.65	−0.791	[−2.966, −2.154]	<0.001	0.643
Onset_ms_EO_R	0.499	107.04	−0.734	[−2.255, −1.536]	<0.001	0.558
Onset_ms_MF_L	0.574	133.79	−0.764	[−2.527, −1.795]	<0.001	0.592
Onset_ms_MF_R	0.474	90.45	−0.693	[−1.767, −1.163]	<0.001	0.502
Onset_ms_TrA/IO region_L	0.513	123.73	−0.749	[−2.190, −1.534]	<0.001	0.606
Onset_ms_TrA/IO region_R	0.240	31.44	−0.514	[−1.366, −0.658]	<0.001	0.274

*p*-values remained significant after Holm–Bonferroni correction for multiple comparisons. ΔR^2^ = change in R-squared; β: standardized regression coefficient; CI represents 95% confidence interval for the unstandardized coefficient (B). EO: External Oblique; LM: Lumbar Multifidus; TrA/IO: Transversus Abdominis/Internal Oblique region. Due to substantial multicollinearity among predictors, regression coefficients should be interpreted as reflecting associative relationships rather than independent effects.

## Data Availability

The datasets generated and/or analyzed during the current study are available from the corresponding author on reasonable request.

## Abbreviations

The following abbreviations are used in this manuscript:

APAs	Anticipatory Postural Adjustments
ASIS	Anterior Superior Iliac Spine
CNS	Central Nervous System
CVA	Craniovertebral Angle
EMG	Electromyography
EO	External Oblique
FHP	Forward Head Posture
IP	Integrated Profile
LM	Lumbar Multifidus
MMT	Manual Muscle Testing
MVIC	Maximal Voluntary Isometric Contraction
NHP	Normal Head Posture
PSM	PostureScreen^®^ Mobile application
RF	Rectus Femoris
RMS	Root Mean Square
sEMG	Surface Electromyography
TrA	Transverse Abdominis Region
TrA/IO	Transverse Abdominis/Internal Oblique Region
